# The transcription factor *Pou4f3* is essential for the survival of postnatal and adult mouse cochlear hair cells and normal hearing

**DOI:** 10.3389/fncel.2024.1369282

**Published:** 2024-03-19

**Authors:** Jarnail Singh, Michelle R. Randle, Bradley J. Walters, Brandon C. Cox

**Affiliations:** ^1^Department of Pharmacology, Southern Illinois University School of Medicine, Springfield, IL, United States; ^2^Department of Otolaryngology-Head and Neck Surgery, University of Mississippi Medical Center, Jackson, MS, United States; ^3^Department of Otolaryngology, Southern Illinois University School of Medicine, Springfield, IL, United States

**Keywords:** Pou4f3, DFNA15, hearing loss, hair cell maturation, hair cell survival

## Abstract

**Introduction:**

Hair cells (HCs) of the cochlea are responsible for sound transduction and hearing perception in mammals. Genetic mutations in the transcription factor *Pou4f3* cause non-syndromic autosomal dominant hearing loss in humans (DFNA15) which varies in the age of onset depending on the individual mutation. Mouse models with germline deletion or mutations in *Pou4f3* have previously demonstrated its critical role in the maturation and survival of cochlear HCs during embryonic development. However, the role of *Pou4f3* in auditory function and in the survival or maintenance of cochlear HCs after birth and during adulthood has not been studied.

**Methods:**

Therefore, using the inducible CreER-loxP system, we deleted *Pou4f3* from mouse cochlear HCs at different postnatal ages, relevant to specific stages of HC maturation and hearing function.

**Results and discussion:**

Elevated auditory brainstem response thresholds and significant HC loss were detected in mice with *Pou4f3* deletion compared to their control littermates, regardless of the age when *Pou4f3* was deleted. However, HC loss occurred more rapidly when *Pou4f3* was deleted from immature HCs. Additionally, HC loss caused by *Pou4f3* deletion did not affect the number of cochlear supporting cells, but caused a delayed loss of spiral ganglion neurons at 4 months after the deletion. In conclusion, *Pou4f3* is necessary for the survival of cochlear HCs and normal hearing at all postnatal ages regardless of their maturation state. Our data also suggest that *Pou4f3* indirectly regulates the survival of spiral ganglion neurons.

## 1 Introduction

Mechanosensory hair cells (HCs) mediate hearing by converting sound waves into electrical signals which are relayed to the brain via spiral ganglion neurons (SGNs). The sensory epithelium of the mammalian cochlea, called the organ of Corti, contains two types of HCs: the inner HCs (IHCs) which act as primary transducers of sound and the outer HCs (OHCs) which act as amplifiers (reviewed in Fettiplace, 2017). Malformation or degeneration of cochlear HCs is one of the most common causes of congenital hearing loss derived from genetic mutations (Egilmez and Kalcioglu, 2016; Korver et al., 2017; Nicolson, 2021). Over 120 genes have been identified that cause non-syndromic hearing loss by autosomal dominant (75%−80%), autosomal recessive (20%), X-linked (2%) or mitochondrial (1%) gene mutations (Sheffield and Smith, 2019; Tollefson et al., 2023).

In humans, the autosomal dominant mutation DFNA15 lies in the transcription factor *Pou4f3* which was first identified in an Israeli Jewish family (Vahava et al., 1998). Since then, various *Pou4f3* mutations have been identified in humans which cause altered DNA binding, cellular localization, and/or stability of the POU4F3 protein (Weiss et al., 2003; Collin et al., 2008; Bai et al., 2020). While patients with DFNA15 are born with normal hearing, they experience progressive hearing loss between 3 and 50 years of age depending on the specific mutation (Vahava et al., 1998; Frydman et al., 2000; Kitano et al., 2017; Lin et al., 2017; Cui et al., 2020).

In loss of function experiments, both *in vitro* and *in vivo, Pou4f3* has been shown to be important for HC development, promoting differentiation, maturation, and survival, which also impacted the innervation and survival of spiral ganglion neurons (SGNs) (Erkman et al., 1996; Xiang et al., 1997, 1998, 2003). POU4F3 protein has also been shown to act as a pioneer factor for opening closed chromatin to allow proper differentiation of HCs during embryonic development (Yu et al., 2021). Mechanistic studies have shown that *Pou4f3* regulates HC survival *in vivo* via its target gene, growth factor independence 1 (*Gfi1*), which directly inhibits the expression of the stress granule protein, CAPRIN-1 (Hertzano et al., 2004; Towers et al., 2011) and indirectly induces anti-apoptotic genes such as *Bcl2* and *Bcl-xL* (Fukada et al., 1996; Catlett-Falcone et al., 1999; Alonzi et al., 2001). Despite the usefulness of these mouse models with germline mutations to define *Pou4f3's* role in newly differentiating HCs during embryonic development, the delayed onset phenotypes observed in humans born with *Pou4f3* mutations is quite different. Thus, using the inducible CreER-loxP system which allows temporal control of gene deletion, we aimed to investigate the role of *Pou4f3* in the postnatal and adult mouse cochlea since HCs are not mature at birth and hearing onset does not occur until ~2 weeks of age in mice (Ehret, 1976).

Using mouse models for conditional deletion, we investigated the role of *Pou4f3* at different stages of cochlear maturation: at birth, at 2 weeks of age, at 4 weeks of age, and at 8 weeks of age. Regardless of the postnatal age when *Pou4f3* was deleted, we observed significant hearing loss and HC loss, but the rate of HC death varied across cochlear turns and was delayed when *Pou4f3* was deleted from mature HCs at 8 weeks of age. However, while supporting cells remained present at normal numbers 4 months after HC loss caused by *Pou4f3* deletion, we observed significant loss of SGNs. Thus, in addition to regulating HC survival throughout the lifespan of the HC, loss of *Pou4f3* from HCs indirectly leads to diminished survival of auditory neurons.

## 2 Materials and methods

### 2.1 Mouse lines

*Atoh1-CreER*™ (Chow et al., 2006) and *Prestin*^*CreERT*2^(Fang et al., 2012) mice were obtained from Dr. Suzanne Baker and Dr. Jian Zuo at St. Jude Children's Research Hospital (Memphis, TN), respectively. *Pou4f3*^*loxP*/*loxP*^ (stock # 10560) (Badea and Nathans, 2011) and *ROSA26*^*CAG*−*loxP*−*stop*−*loxP*−*tdTomato*^ (*ROSA26*^*tdTomato*^) mice (stock # 7914; also called Ai14) (Madisen et al., 2010) were purchased from The Jackson Laboratory (Bar Harbor, ME). All genotyping was performed by Transnetyx, Inc. (Cordova, TN) and mice of both genders were used in the study. All animal work was performed in accordance with approved animal protocols from the Institutional Animal Care and Use Committee at Southern Illinois University School of Medicine.

### 2.2 Drug treatments

CreER recombination was induced at different postnatal ages by intraperitoneal (IP) injections of tamoxifen (Sigma-Aldrich, St. Louis, MO) dissolved in corn oil. Injections of 3 mg/40 g body weight (b.w.) were given 20–24 h apart at postnatal day (P)0 and P1 or at P12 and P13. Adult mice were injected with 9 mg/40 g b.w. tamoxifen for 2 consecutive days (20–24 h between injections) at either 4 or 8 weeks of age. Cre-negative littermates injected with tamoxifen served as controls.

### 2.3 Immunofluorescence

Temporal bones were post-fixed in 4% paraformaldehyde (Polysciences Inc, Warrington, PA) in 10 mM PBS for 2 h at room temperature. Samples from mice that were older than 1 week were decalcified in 120 mM EDTA (Sigma-Aldrich, St. Louis, MO) for 1–5 overnights (depending on the age of mouse at tissue collection) at room temperature (RT) using an end-over-end rotator and the EDTA solution was changed daily. Whole mount dissection and immunostaining was performed as previously described (Montgomery and Cox, 2016) with the one exception that the samples were incubated with signal enhancer (cat #I36933; Life Technologies, Waltham, MA) for 30 min at RT prior to the blocking/permeabilization step. The following primary antibodies were used: mouse anti-beta III tubulin (Tuj1, 1:500, Biolegend, cat # 801201), rabbit anti-myosin VIIa (1:200; cat. #25-6790; Proteus Biosciences, Ramona, CA), mouse IgG1 anti-Pou4f3 (1:300; cat. #sc-81980; Santa Cruz Biotechnology, Dallas, TX), and goat anti-Sox2 (1:400, cat. #sc-17320; Santa Cruz Biotechnology, Dallas, TX). Alexa-conjugated secondary antibodies (Thermo Fisher Scientific, Hampton, NH) were used at 1:1,000 and nuclei were labeled with Hoechst 33342 (1:2,000 in 10 mM PBS, cat. #H3570 Thermo Fisher Scientific, Hampton, NH). tdTomato was visualized using endogenous fluorescence. TUNEL staining was performed using the *In Situ* Cell Death Detection Kit, TMR Red (cat. #12156792910; Roche Applied Science, Indianapolis, IN) following the manufacturer's instructions.

### 2.4 Cryosections

Post-fixed temporal bones were washed three times in 10 mM PBS and decalcified in 120 mM EDTA (Sigma-Aldrich, St. Louis, MO) for 1–2 weeks at room temperature (RT) using an end-over-end rotator. EDTA solution was changed daily. The decalcified cochleae were embedded in optimal cutting temperature (OCT) media (cat #4585, Fisher Healthcare, Houston, TX) in cryo-molds placed on a slurry of dry ice in 70% ethanol. The embedded cochleae were stored at −80°C until sectioning. For analyses of SGNs, mid-modiolar cryo-sections (12 μm thickness) were obtained using an Epredia Microm HM525 NX cryostat (Thermo Fisher Scientific, Hampton, NH) as previously described (Coleman et al., 2009).

### 2.5 Image analysis

Samples were imaged using a Zeiss LSM800 (Oberkochen, Germany) confocal microscope and processed using Zen 2.5 lite software (Oberkochen, Germany). Myosin VIIa-positive HCs and Sox2-positive SCs were manually quantified from two representative 150 μm regions from each cochlear turn (apex, middle, and base) from cochlear whole-mounts. Tuj1-positive neuronal cell bodies from cochlear sections were quantified in a 10,000 μm^2^ region in the middle turn of cochleae from three alternate sections per sample. For quantification, cochlear whole mounts or sections were imaged using a 40× oil immersion objective with a numerical aperture (NA) 1.3 and a resolution of 1,024 × 1,024 pixels. The low magnification imaging of cochlear sections was done using a 10× objective with NA 0.3 and a resolution of 1,024 × 1,024 pixels. The *N* values represent number of mice as only one cochlea per mouse were analyzed for each immunostaining experiment type.

### 2.6 Auditory brainstem response recordings

Mice aged 4 weeks or older were anesthetized with avertin (250–500 mg/kg, IP) and kept on a heating pad at 37°C in a sound attenuated chamber. Auditory brainstem response (ABR) measurements were performed using an Intelligent Hearing System (IHS, Miami, FL) 4964 high frequency system using the left ear of each mouse. Therefore, *N* values represent the number of mice. Subdermal stainless-steel electrodes were inserted at the vertex of the skull, below the pinna of the left ear, and a ground electrode was located at the base of the tail. ABR waveforms were obtained in response to 8, 12, 16, and 22 kHz tones (5 ms tone bursts presented at a rate of 19/s and averaged over 512 presentations) given in 5 dB SPL steps decrements between 80 and 5 dB SPL. ABR thresholds were determined by the lowest sound intensity that produced a visually distinct response in wave I and II as assessed by a researcher who was blinded to the genotypes.

### 2.7 RNA extraction and quantitative real-time PCR

The cochlear portion of temporal bones were collected and snap frozen on dry ice and kept at −80°C until further processed for RNA isolation. Both cochleae from each mouse were pooled for RNA isolation hence *N* values for qPCR analysis represent the number of mice. RNA isolation and real-time quantitative polymerase chain reaction (qPCR) was performed as previously described (McGovern et al., 2018) using SYBR Green (cat #K0391, Thermo Fisher Scientific, Hampton, NH) and a CFX Connect Optics Module (Bio-Rad, Hercules, CA). The primers used for each gene are listed in Supplementary Table 1. Comparison of gene expression levels was determined using the Pfaffl method which includes the primer efficiency in the delta-delta Ct equation (Pfaffl, 2001).

### 2.8 Statistical analysis

**Figure 1:**
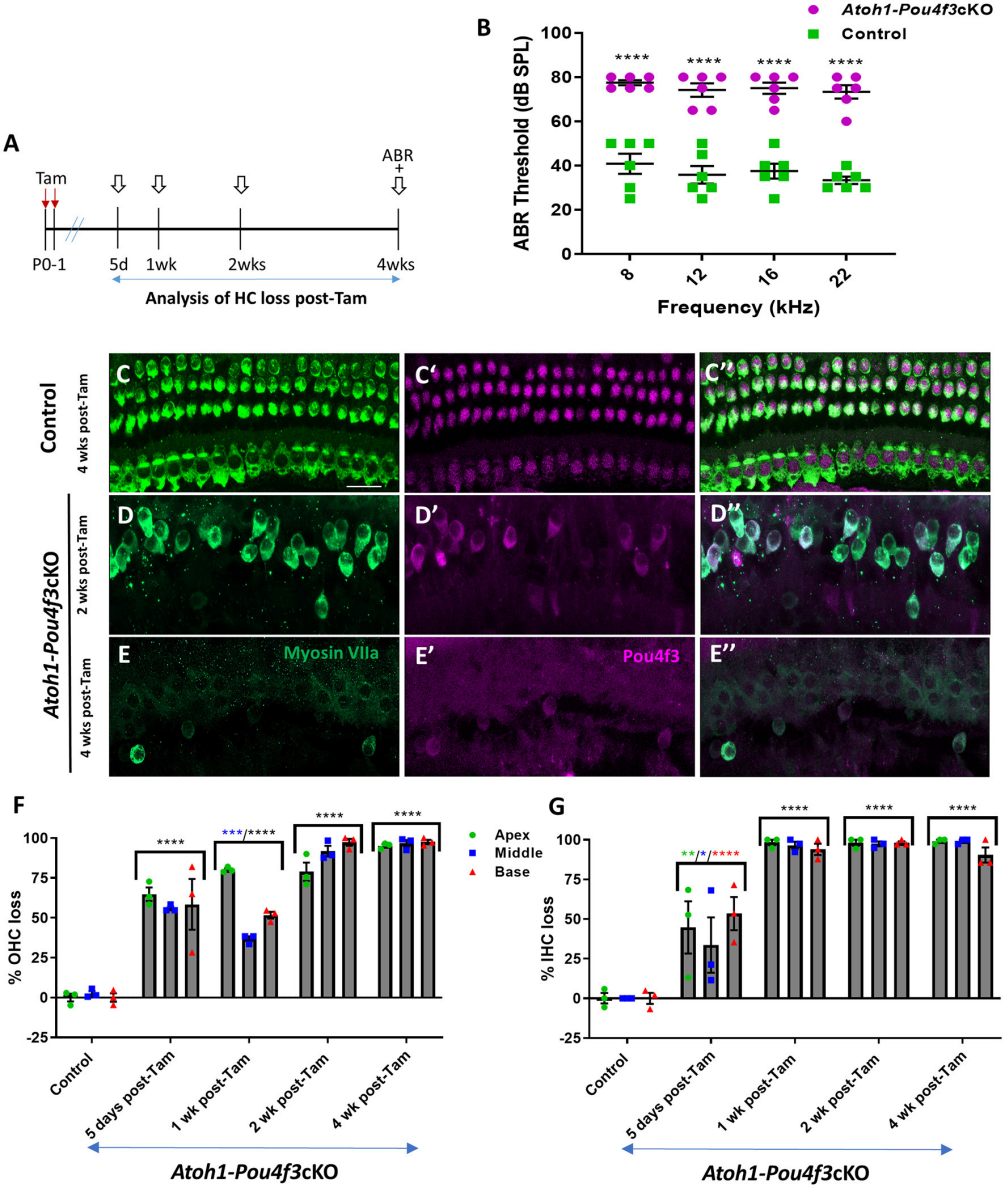
**(A)** Schematic for the experimental design using *Atoh1-Pou4f3*cKO mice to delete *Pou4f3* from both IHCs and OHCs at P0–P1. Open arrows indicate post-tamoxifen (post-Tam) timepoints when HC loss was assessed. **(B)** At 4 weeks after *Pou4f3* deletion, there was a significant elevation in ABR thresholds in *Atoh1-Pou4f3*cKO mice compared to their control littermates at all frequencies tested [*N* = 6; significant main effect of genotype, *F*_(1,40)_ = 302.8, *p* < 0.0001]. Asterisks indicate comparisons between genotypes at each frequency based on a Bonferroni-corrected *post-hoc* test. **(C–E″)** Representative confocal images from control **(C–C″)** and *Atoh1-Pou4f3*cKO **(D–E″)** cochleae. HCs in the control cochleae remained intact (myosin VIIa, green) and had nuclear expression of POU4F3 (magenta). However, many HCs were missing in *Atoh1*-*Pou4f3*cKO cochleae at 2 and 4 weeks after *Pou4f3* deletion and most of the remaining HCs exhibited POU4F3 immunoreactivity in their cytoplasm. Quantification of OHC **(F)** and IHC **(G)** loss in control and *Atoh1-Pou4f3*cKO cochleae (*N* = 3) between 5 days and 4 weeks post-Tam. For OHCs there was a significant main effect of time [*F*_(4,10)_ = 142.2, *p* < 0.0001] and interaction between time and cochlear turn [*F*_(8,20)_ = 6.697, *p* = 0.0003]. For IHCs there was a significant main effect of time [*F*_(4,10)_ = 56.95, *p* < 0.0001]. In **(F)** and **(G)**, differences from control within each cochlear turn are indicated by the asterisks based on a Tukey's-corrected *post-hoc* test. Green asterisks are *p* values for the apical turn, blue asterisks are *p* values for the middle turn, and red asterisks are *p* values for the basal turn. Black asterisks were used when the *p* value was the same for two or three turns. Comparisons across time post-Tam and across cochlear turns within the same genotype are presented in Supplementary Tables 2A,B. Data are presented as mean ± SEM. *p < 0.05, **p < 0.01, ***p < 0.001 and ****p < 0.0001. Scale bar = 20 μm. *Pou4f3* deletion from immature HCs at birth causes elevated ABR thresholds and progressive HC loss.

**Figure 2:**
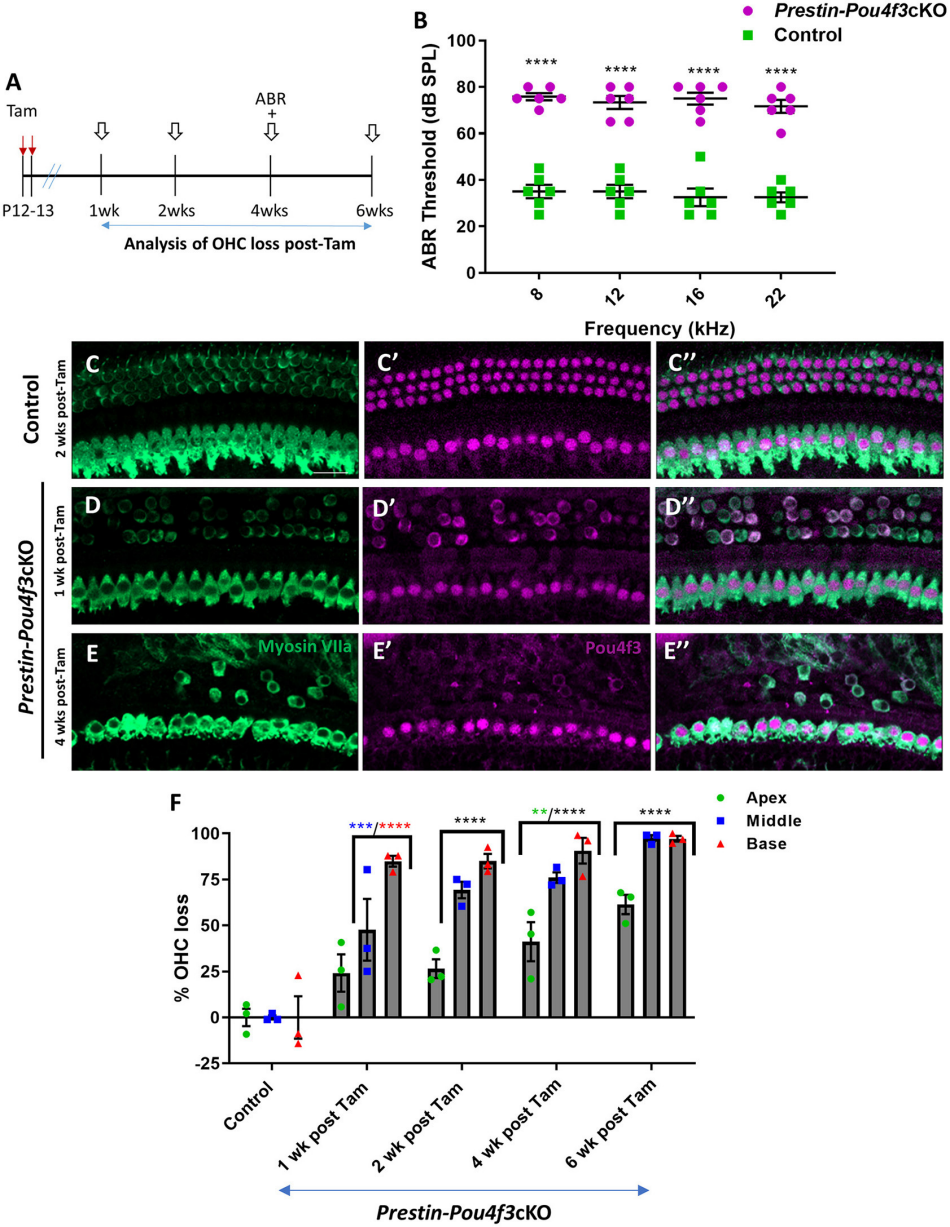
*Pou4f3* deletion from OHCs at hearing onset causes elevated ABR thresholds and significant OHC loss. **(A)** Schematic for the experimental design using *Prestin-Pou4f3*cKO mice to delete *Pou4f3* from OHCs at 2 weeks of age. Open arrows indicate post-tamoxifen (post-Tam) timepoints when OHC loss was assessed. **(B)** At 4 weeks (wks) after *Pou4f3* deletion, there was a significant elevation in ABR thresholds in *Prestin-Pou4f3*cKO mice compared to their control littermates at all frequencies tested [*N* = 6, significant main effect of genotype, *F*_(1,40)_ = 428.1, *p* < 0.0001]. Asterisks indicate comparisons between genotypes at each frequency based on a Bonferroni corrected *post-hoc* test. **(C–E″)** Representative confocal images from control **(C–C″)** and *Prestin-Pou4f3*cKO **(D–E″)** cochleae. HCs in the control cochleae and IHCs in the *Prestin*-*Pou4f*3cKO cochleae remained intact (myosin VIIa, green) and had nuclear expression of POU4F3 (magenta). However, many OHCs were missing in *Prestin*-*Pou4f*3cKO cochleae at 1 and 4 weeks after *Pou4f3* deletion. Most of the remaining OHCs at 1 week post-Tam expressed POU4F3 in their cytoplasm. **(F)** Quantification of OHC loss in control and *Prestin-Pou4f3*cKO cochleae (*N* = 3) between 1 and 6 weeks post-Tam. There was a significant main effect of cochlear turn [*F*_(2,20)_ = 43.81, *p* < 0.0001]; time [*F*_(4,10)_ = 49.21, *p* < 0.0001]; and an interaction between time and cochlear turn [*F*_(8,20)_ = 3.832, *p* = 0.0070]. Differences from control within each cochlear turn are indicated by the asterisks based on a Tukey's-corrected *post-hoc* test. Green asterisks are *p* values for the apical turn, blue asterisks are *p* values for the middle turn, and red asterisks are *p* values for the basal turn. Black asterisks were used when the *p* value was the same for two or three turns. Data are presented as mean ± SEM. **p < 0.01, ***p < 0.001, and ****p < 0.0001. Comparisons across time post-Tam and across cochlear turns within the same genotype are presented in Supplementary Table 3. Scale bar =20 μm.

**Figure 3:**
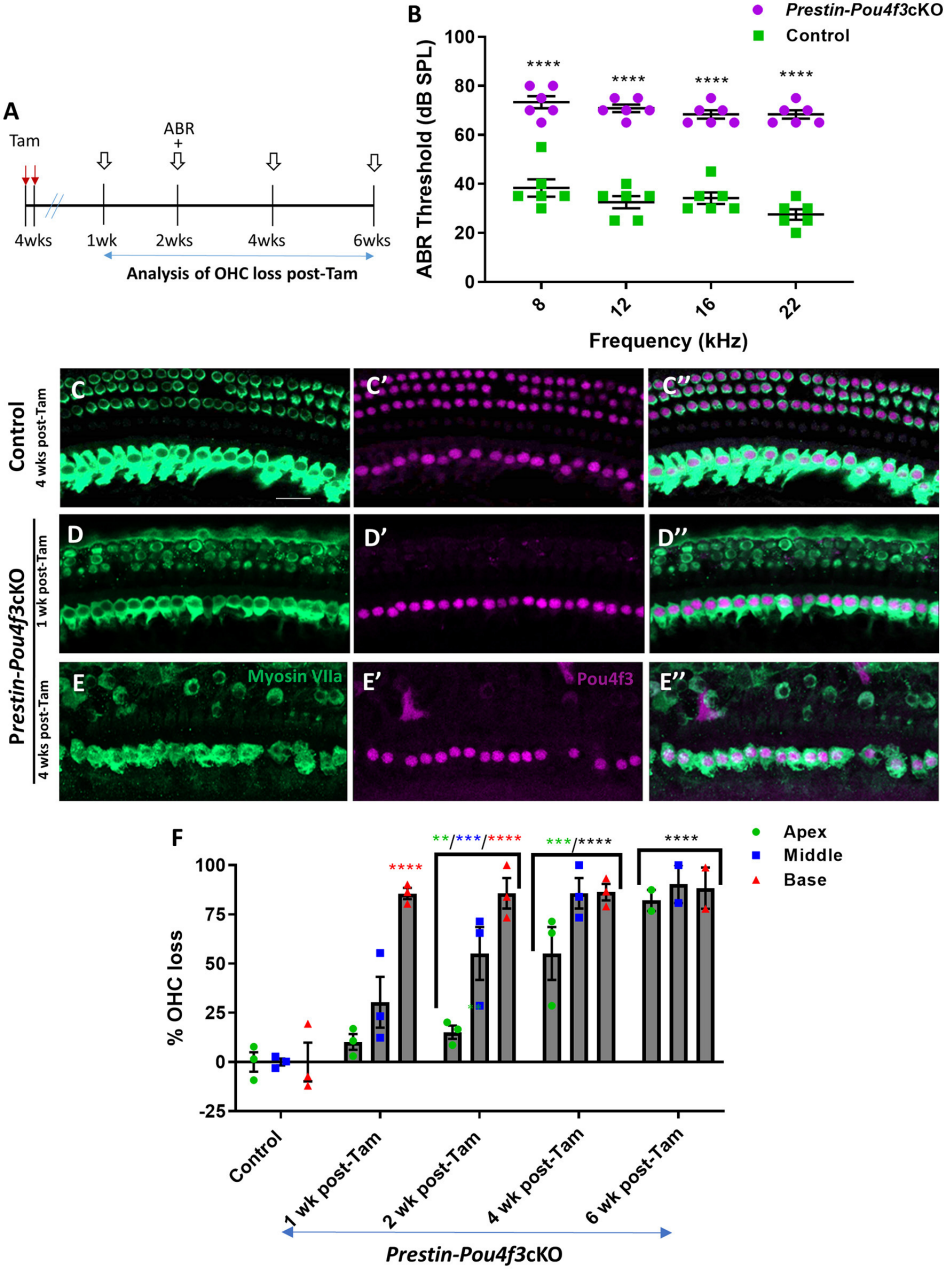
*Pou4f3* deletion from OHCs once hearing is mature causes elevated ABR thresholds and progressive OHC loss. **(A)** Schematic for the experimental design using *Prestin-Pou4f3*cKO mice to delete *Pou4f3* from OHCs at 4 weeks (wks) of age. Open arrows indicate post-tamoxifen (post-Tam) timepoints when OHC loss was assessed. **(B)** At 2 weeks after *Pou4f3* deletion, there was a significant elevation in ABR thresholds in *Prestin-Pou4f3*cKO mice compared to their control littermates at all frequencies tested [*N* = 6; significant main effect of genotype, *F*_(1,10)_ = 355.2, *p* < 0.0001 and frequency *F*_(3,30)_ = 4.539, *p* = 0.0097]. Asterisks indicate comparisons between genotypes at each frequency based on a Bonferroni-corrected *post-hoc* test. **(C–E″)** Representative confocal images from control **(C–C″)** and *Prestin-Pou4f3*cKO **(D–E″)** cochleae. HCs in the control cochleae and IHCs in the *Prestin*-*Pou4f*3cKO cochleae remained intact (myosin VIIa, green) and had nuclear expression of POU4F3 (magenta). Analysis at 1 and 4 weeks after *Pou4f3* deletion showed progressive loss of OHCs in *Prestin*-*Pou4f*3cKO cochleae over time. **(F)** Quantification of OHC loss in control and *Prestin-Pou4f3*cKO cochleae (*N* = 3) between 1 and 6 weeks post-Tam. There was a significant main effect of time [*F*_(4,9)_ =38.51, *p* < 0.0001]; cochlear turn [*F*_(2,18)_ = 25.62, *p* < 0.0001]; and an interaction between time and cochlear turn [*F*_(8,18)_ = 5.625, *p* = 0.0011]. Differences from control within each cochlear turn are indicated by the asterisks based on a Tukey's-corrected *post-hoc* test. Green asterisks are *p* values for the apical turn, blue asterisks are *p* values for the middle turn, and red asterisks are *p* values for the basal turn. Black asterisks were used when the *p* value was the same for two or three turns. Data are presented as mean ± SEM. **p < 0.01, ***p < 0.001 and ****p < 0.0001. Comparisons across time post-Tam and across cochlear turns within the same genotype are presented in Supplementary Table 4. Scale bar =20 μm.

**Figure 4:**
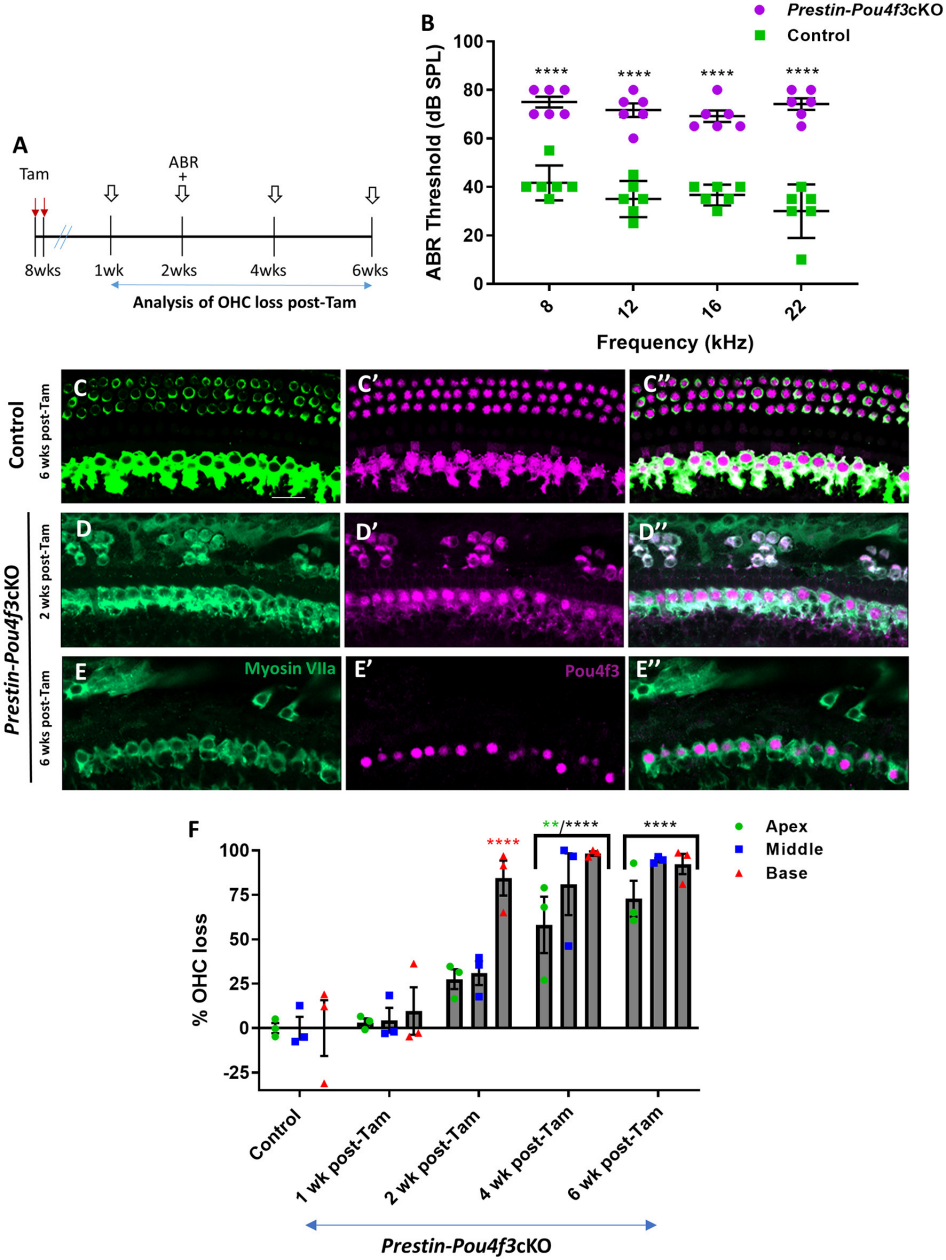
*Pou4f3* deletion from OHCs in sexually mature mice leads to elevated ABR thresholds and progressive OHC loss. **(A)** Schematic for the experimental design using *Prestin-Pou4f3*cKO mice to delete *Pou4f3* from OHCs at 8 weeks (wks) of age. Open arrows indicate post-tamoxifen (post-Tam) timepoints when OHC loss was assessed. **(B)** At 2 weeks after *Pou4f3* deletion, there was a significant elevation in ABR thresholds in *Prestin-Pou4f3*cKO mice compared to their control littermates at all frequencies tested [*N* = 6; significant main effect of genotype, *F*_(1,40)_ = 350.4, *p* < 0.0001]. Asterisks indicate comparisons between genotypes at each frequency based on a Bonferroni-corrected *post-hoc* test. **(C–E″)** Representative confocal images from control **(C–C″)** and *Prestin-Pou4f3*cKO **(D–E″)** cochleae. HCs in the control cochleae and IHCs in the *Prestin*-*Pou4f*3cKO cochleae remained intact (myosin VIIa, green) and had nuclear expression of POU4F3 (magenta). Analysis at 2 and 6 weeks after *Pou4f3* deletion showed progressive loss of OHCs in *Prestin* Cre-*Pou4f*3cKO cochleae over time. The remaining OHCs at 2 weeks post-Tam expressed POU4F3 in their cytoplasm. **(F)** Quantification of OHC loss in control and *Prestin-Pou4f3*cKO cochleae (*N* = 3) between 1 and 6 weeks post-Tam. There was a significant main effect of time [*F*_(4,10)_ = 29.01, *p* < 0.0001]; cochlear turn [*F*_(2,20)_ = 13.88, *p* = 0.0002]; and an interaction between time and cochlear turn [*F*_(8,20)_ = 3.68, *p* = 0.0085]. Differences from control within each cochlear turn are indicated by the asterisks based on a Tukey's-corrected *post-hoc* test. Green asterisks are *p* values for the apical turn and red asterisks are *p* values for the basal turn. Black asterisks were used when the *p* value was the same for two or three turns. Data are presented as mean ± SEM. **p < 0.01 and ****p < 0.0001. Comparisons across time post-Tam and across cochlear turns within the same genotype are presented in Supplementary Table 5. Scale bar = 20 μm.

All data are presented as mean ± SEM. Comparisons of ABR thresholds between genotypes and across frequencies were done using a two-way ANOVA followed by Bonferroni-corrected *post-hoc* testing at each frequency. HC counts from control samples across time for each dataset were tested for similarity using a one-way ANOVA which revealed no significant differences in HC numbers across controls. Thus, we used the mean number of HCs from the latest timepoint assessed for each age of *Pou4f3* deletion as the control samples in Figures 1–4 and to calculate the percentage of HC loss. Percent HC loss was calculated by subtracting the HC counts from an individual sample (control or *Pou4f3* cKO) from the mean HC number of the control group (*N* = 3, from the latest timepoint assessed for each age of *Pou4f3* deletion) and dividing that number by the mean HC number of the control group. The result was then converted to a percentage. For each age of *Pou4f3* deletion, a two-way ANOVA was used to assess HC loss where control samples were treated as a pseudo-pre-injection timepoint. This allowed for analysis of the main questions of interest (effect of *Pou4f3* deletion, effect of time post-injection, and effect of cochlear turn) without having to test three-way interaction terms which were not the focus of these studies. Significant main effects were followed by two Tukey's *post-hoc* tests. One *post-hoc* test compared each timepoint with control as well as to other timepoints, but within each cochlear turn and the second *post-hoc* test compared cochlear turns within each timepoint. Note that IHCs and OHCs were compared separately for the neonatal deletion of *Pou4f3* in Figure 1. The number of SCs were compared across genotype and across cochlear turn using a two-way ANOVA followed by Sidak-corrected *post-hoc* comparisons. The percentage of SGN loss was calculated in a similar manner as percent HC loss using the mean SGN number from control samples as the denominator. The percentage of SGN loss was compared across genotypes using a two-way ANOVA followed by Sidak's *post-hoc* test. qPCR data are presented in the graphs as fold change from control samples and each gene was compared to its own control using a Student's *t*-test. Statistical analyses were conducted using GraphPad Prism 7.0 software (La Jolla, CA).

## 3 Results

### 3.1 *Pou4f3* is required for the survival of immature cochlear HCs in newborn mice

To delete *Pou4f3* from HCs at birth, we used *Atoh1-CreER*^*TM*^ mice which show CreER activity in the majority of both IHCs and OHCs after tamoxifen induction at P0 and P1 (Chow et al., 2006; Weber et al., 2008; Cox et al., 2014). We validated this expression pattern using *Rosa26*^*tdTomato*^reporter mice, and found that 98%−100% of OHCs expressed tdTomato (100% ± 0% in apex, 98.5% ± 1.5% in middle, and 99.3 ± 0.7% in basal turn, *N* = 4, Supplementary Figure 1) and 92–100% of IHCs expressed tdTomato (100% ± 0% in apex, and 93.0% ± 6.2% in middle, and 93.7% ± 5.4% in basal turn, *N* = 4, Supplementary Figure 1).

HCs in the mouse cochleae are immature at birth and undergo various morphological and electrophysiological changes during the first postnatal weeks (Anniko, 1983; Eatock and Hurley, 2003). To determine the role of *Pou4f3* in immature HCs, we generated *Atoh1-CreER*^*TM*^*:Pou*4*f*3^*loxP*/loxP^ mice (referred to hereafter as *Atoh1-Pou4f3*cKO) and injected them with tamoxifen at P0 and P1, followed by assessment of auditory function using auditory brainstem response (ABR) measurements at 4 weeks of age (Figure 1A). Controls were CreER-negative littermates which contained the *Pou*4*f*3^*loxP*/loxP^ allele and also received tamoxifen. Compared to controls, *Atoh1-Pou4f3*cKO mice had significant ABR threshold elevations (~35–40 dB SPL) at all four frequencies tested (Figure 1B). Analysis of the cochleae showed rapid and progressive loss of both IHCs and OHCs in *Atoh1-Pou4f3*cKO mice (Figures 1D–G) compared to controls (Figures 1C–C″,F–G) which had the normal pattern of four rows of HCs with POU4F3 present in all HC nuclei (Figures 1C–C″). In *Atoh1-Pou4f3*cKO mice, OHC loss was observed as early as 5 days post-tamoxifen with more than 50% loss in all cochlear turns (64.8 ± 4.2% in apex; 55.9% ± 1.2% in middle, and 58.4% ± 15.9% in base, *N* = 3), which progressed to >95% OHC loss by 4 weeks post-tamoxifen (95.2% ± 1.0% in apex, 96.7% ± 2.1% in middle, and 97.5% ± 1.3% in base, *N* = 3; Figure 1F, Supplementary Table 2A). IHC loss was also observed at 5 days post-tamoxifen in *Atoh1-Pou4f3*cKO mice, but IHCs died at a faster rate than OHCs with ~94%−98% loss by 1 week post-tamoxifen (98.2 ± 1.8% in apex; 96.6 ± 2.3% in middle; 93.9% ± 3.5% in base, *N* = 3; Figure 1G, Supplementary Table 2B).

### 3.2 *Pou4f3* is required for the survival of OHCs during hearing onset in juvenile mice

HC maturation occurs over the first two postnatal weeks leading to the onset of hearing in mice at ~P12–14 (Ehret, 1976). Since *Atoh1* expression is down-regulated within the first postnatal week, we instead used *Prestin*^*CreERT*2^mice which specifically target OHCs of the cochlea (Fang et al., 2012; Cox et al., 2014). We validated the expression pattern of *Prestin*^*CreERT*2^ using *Rosa*26^*tdTomat*o^ reporter mice and found that >90% of OHCs expressed tdTomato in all three turns of the cochlea after tamoxifen injections at P12 and P13, 4 weeks of age, or 8 weeks of age (Supplementary Figure 2).

To determine whether mature OHCs still require *Pou4f3* for survival, we generated *Prestin*^*CreERT*2^*::Pou4f3*^*loxP*/*loxP*^ mice (*Prestin-Pou4f3*cKO), injected them with tamoxifen at P12 and P13 (juvenile age), and performed similar assessments as outlined above (Figure 2A). Controls were CreER-negative littermates which contained the *Pou*4*f*3^*loxP*/loxP^ allele and also received tamoxifen. *Prestin-Pou4f3*cKO mice also showed significant ABR threshold elevations (~33–43 dB SPL) at all four frequencies tested compared to CreER-negative control littermates when tested at 4 weeks after deletion of *Pou4f3* (Figure 2B). Compared to controls, we observed progressive loss of OHCs in all three turns of *Prestin-Pou4f3*cKO cochleae between 1 and 6 weeks post-tamoxifen (Figures 2C–F). However, IHCs remained intact with all expressing nuclear POU4f3 (Figures 2D,E″). This was expected since *Prestin*^*CreERT*2^ targets OHCs exclusively. Comparison of OHC loss across cochlear turns in *Prestin-Pou4f3*cKO mice at 1 week post-tamoxifen showed greater loss in the basal turn (84.9 ± 2.9%) than the middle (47.6 ± 16.8%) and apical turns (24.2 ± 10.1%; Figure 2F, Supplementary Table 3). Yet, OHC loss progressed in all cochlear turns during the following weeks resulting in almost complete absence of OHCs in the middle and basal turns (97.5 ± 1.7% in middle, and 97.2 ± 1.5% in base), but only ~60% loss in the apical turn (61.5 ± 5.3%) at 6 weeks post-tamoxifen (Figure 2F, Supplementary Table 3).

### 3.3 *Pou4f3* is required for the survival of OHCs in adult mice

Next, we investigated the role of *Pou4f3* in OHCs when mice have mature hearing (at 4 weeks of age), or after they were sexually mature at (8 weeks of age) (reviewed in Ohlemiller et al., 2016). For both experiments, we again used *Prestin-Pou4f3*cKO mice to delete *Pou4f3* from OHCs and performed ABR measurements and histological analyses of the cochlea at multiple timepoints (Figures 3A, 4A). Controls were CreER-negative littermates which contained the *Pou*4*f*3^*loxP*/loxP^ allele and also received tamoxifen. After *Pou4f3* deletion at 4 weeks of age, we observed significant elevation of ABR thresholds in *Prestin-Pou4f3*cKO mice at all frequencies tested at 2 weeks post-tamoxifen compared to their control littermates (Figure 3B). One week after *Pou4f3* deletion, OHC numbers in the apical and middle turns of *Prestin-Pou4f3*cKO cochleae were not different from controls, but there was significant and robust OHC loss (85.6 ± 2.9%) in the basal turn (Figures 3C–F). Two weeks after *Pou4f3* deletion, OHC loss was significantly higher in all cochlear turns compared to controls with the highest loss observed in the basal turn (85.8 ± 7.7%) followed by the middle turn (55.2 ± 13.4%), but there was only a mild loss (15.1 ± 3.4%) in the apex. OHC loss in apical and middle turns continued to progress over time with the middle turn reaching >80% loss (85.8 ± 7.7%) at 4 weeks post-tamoxifen and the apical turn reaching similar loss level (82.1 ± 5.4%) at 6 weeks post-tamoxifen (Figure 3F, Supplementary Table 4).

Finally, we deleted *Pou4f3* from OHCs at 8 weeks of age using *Prestin-Pou4f3*cKO mice to assess the role of *Pou4f3* in sexually mature mice and to test whether *Pou4f3* is required for OHC survival throughout the lifespan (Figure 4A). Similar to *Pou4f3* deletion at all other ages, we observed significant elevation in ABR thresholds in *Prestin-Pou4f3*cKO mice compared to control mice at 2 weeks post-tamoxifen at all frequencies tested (Figure 4B). However, OHC loss was delayed compared to *Pou4f3* deletion at other ages (Figures 4D–F). There was no significant difference in the number of OHCs in *Prestin-Pou4f3cKO* cochleae compared to controls at 1 week post-tamoxifen. However, at 2 weeks post-tamoxifen a majority of the OHCs were missing in the basal turn (84.5 ± 9.8%; Figure 4F). By 4 weeks post-tamoxifen, OHC loss progressed to the middle (81.0 ± 17.4%) and apical (58.1 ± 15.8%) turns as well (Figure 4F, Supplementary Table 5).

### 3.4 *Pou4f3* deletion from HCs causes cell death by apoptosis

**Figure 5:**
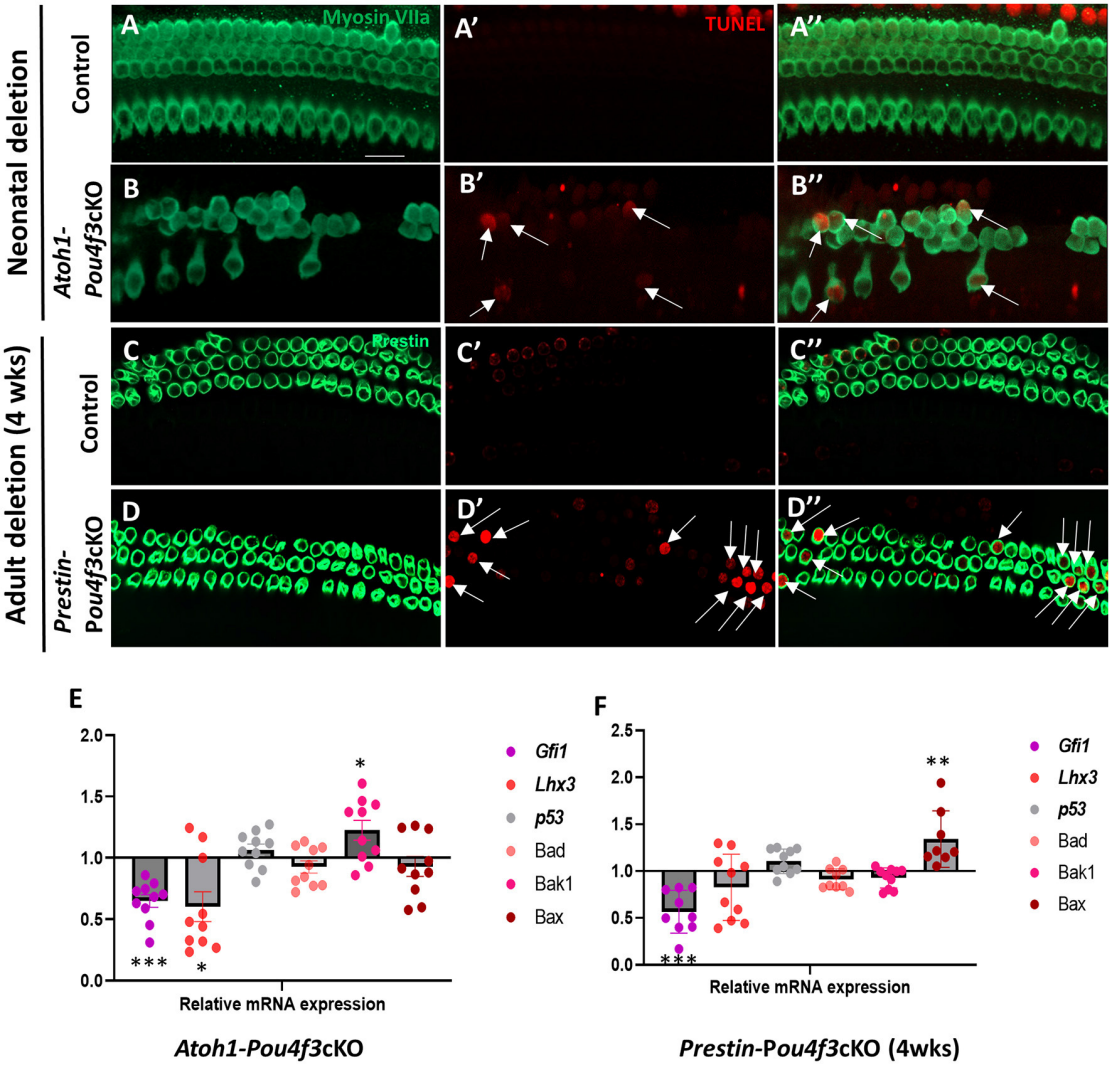
*Pou4f3* deletion at both neonatal and adult ages causes HC death by apoptosis. **(A–D″)** Representative confocal images from control **(A–A″, C–C″)** and *Atoh1-Pou4f3*cKO **(B–B″)** or *Prestin-Pou4f3*cKO **(D–D″)** cochleae 5 days after deletion of *Pou4f3* at P0/P1 or one week after deletion of *Pou4f3* at 4 weeks (wks) of age respectively. IHCs and OHCs were stained using myosin VIIa [green, **(A–B″)**] or the OHC-specific marker prestin [green, **(C–D″)**]. TUNEL staining (red) was used to identify apoptotic cells. No TUNEL staining was observed in control cochleae. However, many IHCs and OHCs from Atoh*1-Pou4f3*cKO cochleae and OHCs from *Prestin-Pou4f3*cKO cochleae were TUNEL-positive (arrows). **(E, F)** Quantitative real-time PCR analysis of the mRNA transcripts for the *Pou4f3* target genes, *Gfi1* and *Lhx3*, as well as the pro-apoptotic genes, *p53, Bad, Bak1* and *Bax*. Samples were analyzed 5 days after deletion of *Pou4f3* at P0/P1 using *Atoh1-Pou4f3*cKO mice or 1 week after deletion of *Pou4f3* at 4 weeks of age using *Prestin-Pou4f3*cKO mice. Data are expressed as fold change (mean ± SEM) from control. Each gene was compared to its own control using a Student's *t*-test. *N* = 8–10. *p < 0.05, **p < 0.01, ***p < 0.001. Scale bar = 20 μm.

Next, we sought to investigate the mechanism of cochlear HC death caused by *Pou4f3* deletion. Using cochleae from *Atoh1-Pou4f3*cKO mice injected with tamoxifen at P0 and P1 and *Prestin-Pou4f3*cKO mice injected with tamoxifen at 4 weeks of age, we performed the terminal deoxynucleotidyl transferase dUTP nick end labeling (TUNEL) assay to detect cells undergoing apoptosis. In *Atoh1-Pou4f3*cKO samples where CreER targeted both OHCs and IHCs, we observed TUNEL-positive HCs of both types at 5 days post-tamoxifen (Figures 5B′,B″). Similarly, 1 week after *Pou4f3* deletion, we observed many TUNEL-positive OHCs in *Prestin-Pou4f3*cKO cochleae (Figures 5D′,D″). No TUNEL-positive HCs were observed in any of the control samples (Figures 5A–A″,C–C″).

To further investigate potential mediators of cell death in response to the deletion of *Pou4f3*, we performed real-time qPCR of pro-apoptotic genes and two known POU4F3 target genes using the same mouse models and collection timepoints as used for the TUNEL assay (*Atoh1-Pou4f3*cKO mice were injected with tamoxifen at P0 and P1 and samples collected 5 days post-tamoxifen; *Prestin-Pou4f3*cKO mice were injected with tamoxifen at 4 weeks of age and samples collected at 1-week post-tamoxifen). After deletion of *Pou4f3* we observed the downregulation of two downstream targets of *Pou4f3, Gfi1* (*p* = 0.0003 for *Atoh1-Pou4f3*cKO, and *p* = 0.0009 for *Prestin-Pou4f3*cKO at 4 weeks, *N* = 9–10) and *Lhx3* (*p* = 0.0427 for *Atoh1-Pou4f3*cKO, *N* = 10; Figures 5E,F) (Hertzano et al., 2004, 2007). Five days after neonatal *Pou4f3* deletion using *Atoh1-Pou4f3cKO* mice, we also observed a significant upregulation of the pro-apoptotic gene, *Bak1* (*p* = 0.0398; Figure 5E, *N* = 9–10). One week after *Pou4f3* deletion at 4 weeks of age using *Prestin-Pou4f3*cKO mice, we observed upregulation of the pro-apoptotic gene *Bax* (*p* = 0.0059, *N* = 8). Together these data suggest that HCs undergo apoptotic cell death after deletion of *Pou4f3*.

### 3.5 HC loss caused by *Pou4f3* deletion does not affect the survival of supporting cells, but causes SGN loss

**Figure 6:**
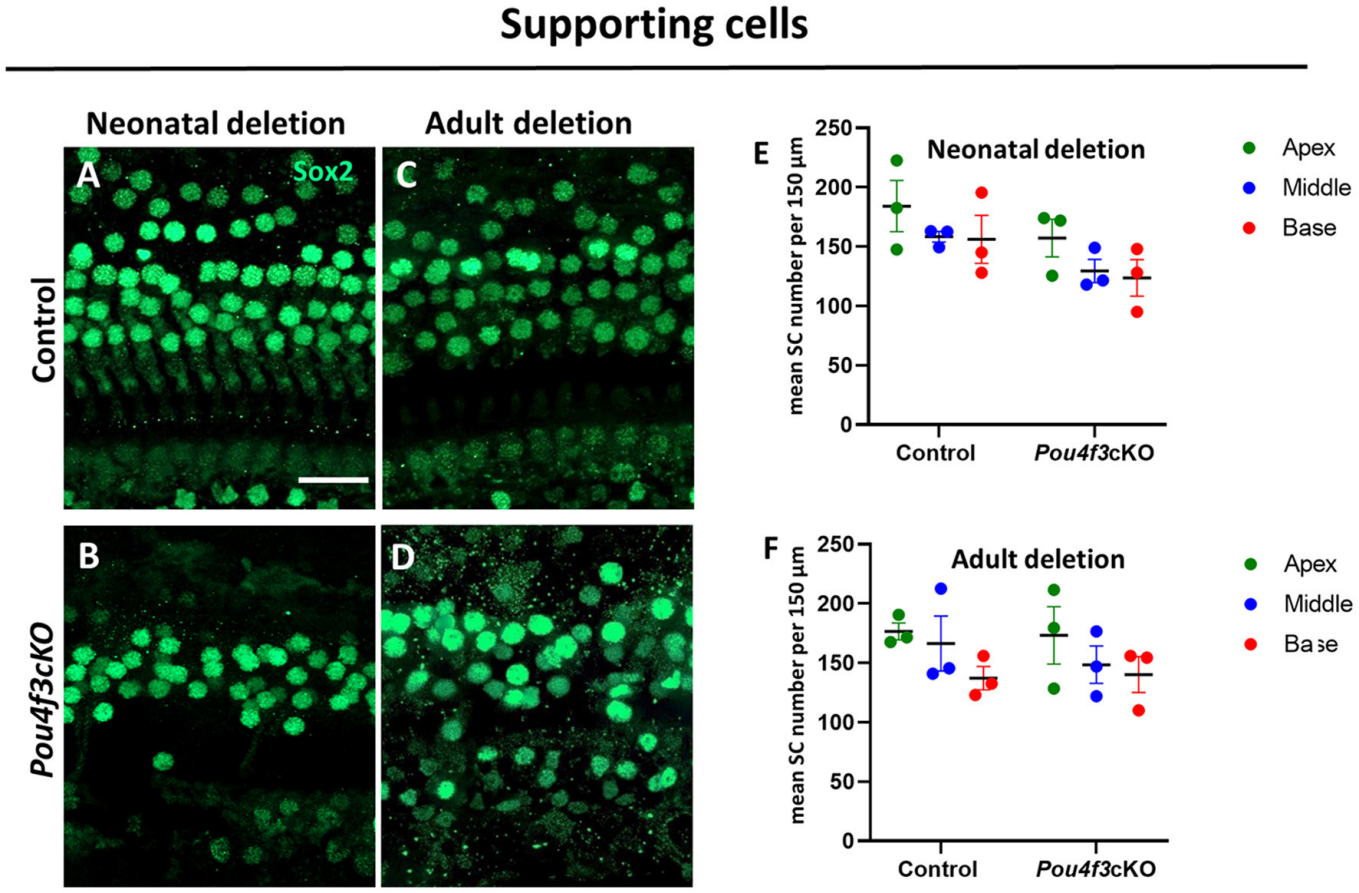
HC loss caused by *Pou4f3* deletion does not affect the survival of SCs. **(A–D)** Representative confocal images of the middle turn from control **(A, C)** and cochleae with *Pou4f3* deletion at P0 using *Atoh1-Pou4f3*cKO mice **(B)** or at 8 weeks using *Prestin-Pou4f3*cKO mice **(D)**. All samples were analyzed at 4 months post-tamoxifen. **(E, F)** Quantification of SOX2-positive SC nuclei in each cochlear turn for control and after *Pou4f3* deletion at P0/P1 **(E)** or 8 weeks of age **(F)**. SCs numbers were compared to control within the same cochlear turn using a two-way ANOVA followed by Sidak's *post-hoc* test. There was a significant main effect of genotype [*F*_(1,6)_ = 12.93, *p* = 0.0114] when *Pou4f3* was deleted at P0/P1, but there were no main effects when *Pou4f3* was deleted at 8 weeks of age and no significant differences in the *post-hoc* tests for either age of *Pou4f3* deletion. *N* = 3. Scale bar = 20 μm.

Previous studies have shown that HC loss can produce secondary effects on the organ of Corti which includes loss of supporting cells and/or delayed SGN loss (Leake et al., 1997; Izumikawa et al., 2008; Kujawa and Liberman, 2009; Barclay et al., 2011; Taylor et al., 2012; Yu et al., 2014). In addition, previous studies showed a loss of SGNs when *Pou4f3* was deleted from the germline (Xiang et al., 2003; Pauley et al., 2008). Here, we investigated whether HC loss caused by *Pou4f3* deletion impacts these two cell types. Using *Atoh1-Pou4f3*cKO mice injected with tamoxifen at P0 and P1 and *Prestin-Pou4f3*cKO mice injected with tamoxifen at 8 weeks of age, we collected samples at 16 weeks post-tamoxifen and performed immunostaining to detect SOX2, a supporting cell-specific marker in the mature cochlea (Figures 6A–D) (Hume et al., 2007). Quantification of SOX2-positive supporting cells revealed no significant differences from control samples for both ages of *Pou4f3* deletion (Figures 6E,F).

**Figure 7:**
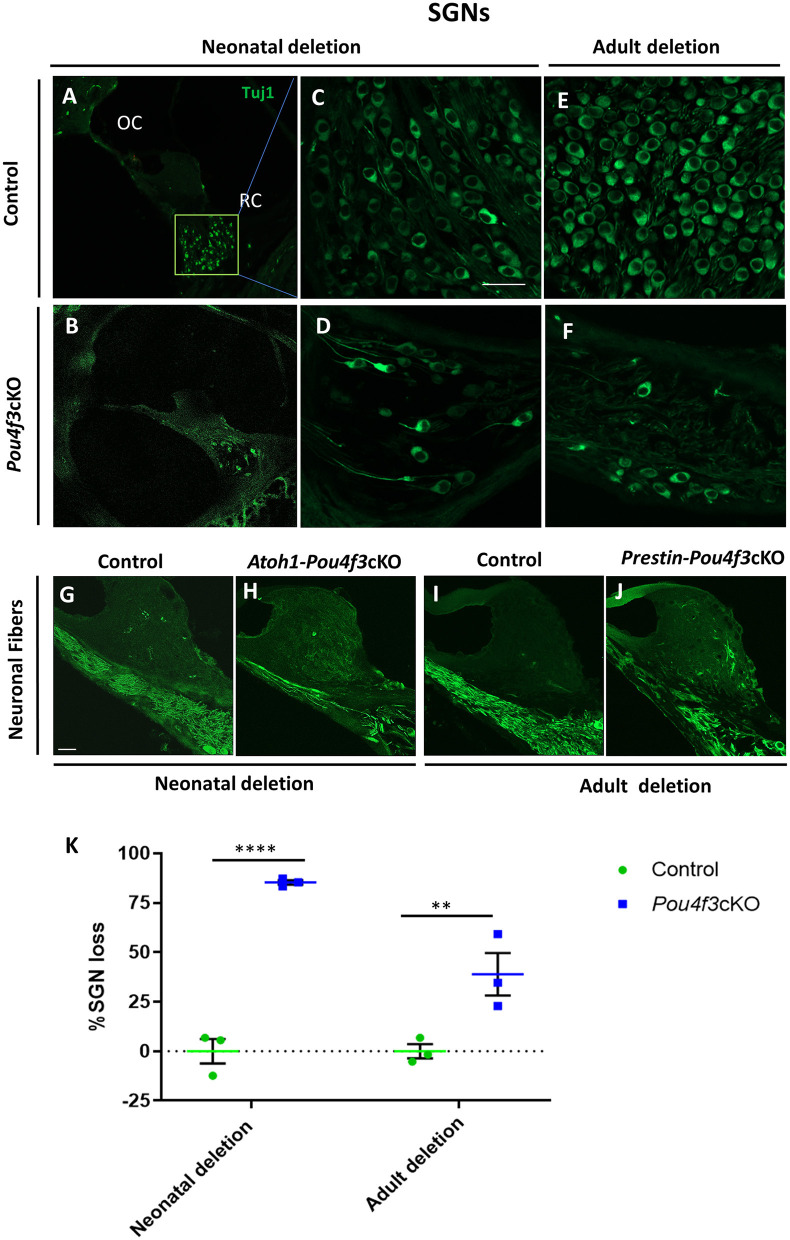
*Pou4f3* deletion affects SGN survival in the long term. Representative confocal images of mid-modiolar cryosections from control **(A, C, E)** and cochleae with *Pou4f3* deletion at P0/P1 using *Atoh1-Pou4f3*cKO mice **(B, D)** or at 8 weeks using *Prestin-Pou4f3*cKO mice **(F)**. All samples were analyzed at 4 months post-tamoxifen. SGN cell bodies were identified using Tuj1 immunostaining (green) in the middle turn of each cochleae. Representative images of neuronal fibers projecting to the organ of Corti from control **(G, I)**, *Atoh1 Cre-Pou4f3*cKO **(H)** and *Prestin Cre-Pou4f3*cKO **(J)** cochleae after deletion of *Pou4f3* at neonatal or adult ages. **(K)** Quantification of Tuj1-positive SGNs after deletion of *Pou4f3* at neonatal and adult ages. *N* = 3. There was a significant main effect of genotype [*F*_(1,4)_ = 78.34, *p* = 0.0009]; age of *Pou4f3* deletion [*F*_(1,4)_ = 15.84, *p* = 0.0164]; and an interaction between genotype and age of *Pou4f3* deletion [*F*_(1,4)_ = 15.84, *p* = 0.0164]. Differences in the percentage of SGN loss between control samples and the respective age of deletion are indicated by the asterisks based on a Sidak's *post-hoc* test. OC, organ of Corti; RC, Rosenthal's canal. Scale bar = 20 μm.

To investigate the impact of *Pou4f3* deletion from HCs on the SGNs, we performed mid-modiolar sectioning of the temporal bones from the same two mouse models at 16 weeks (or 4 months) post-tamoxifen and stained them with the neuron specific anti-Tuj1 antibody (Lee et al., 1990; Barclay et al., 2011; Sun et al., 2018). While the CreER-negative control samples showed normal densities of SGN cell bodies (Figures 7A,C,E), cochleae from mice where *Pou4f3* was deleted from both IHCs and OHCs at P0/P1, had 85.3 ± 1.1% loss of SGN cell bodies (Figures 7B,D,K). However, when the deletion was OHC-specific and occurred at 8 weeks of age, there was a 38.9 ± 10.7% loss of SGNs (Figures 7F,K). Additionally, we observed reduction in the neuronal fibers in *Pou4f3cKO* cochleae (Figures 7H,J) compared to control cochleae (Figures 7G,I) at both ages of *Pou4f3* deletion.

## 4 Discussion

The present study demonstrates that the transcription factor *Pou4f3* is essential for HC survival during postnatal maturation and in adulthood, which is consistent with the data from studies using germline deletion of *Pou4f3* (Erkman et al., 1996; Xiang et al., 1997, 1998, 2003). After *Pou4f3* deletion, HC death occurred via apoptosis as evidenced by TUNEL staining and upregulation of the pro-apoptotic genes, *Bak1* and *Bax*. HC loss after *Pou4f3* deletion did not seem to affect the number of surviving supporting cells, but small changes may not have been detected due to our sample size. This result is similar to other studies where the selective loss of cochlear HCs did not influence the survival of the adjacent supporting cells (Oesterle et al., 2008; Tong et al., 2015). However, the numbers of SGNs was reduced at 4 months after *Pou4f3* deletion, with a larger negative impact when *Pou4f3* was deleted from neonatal HCs.

OHCs are generally thought to be more susceptible to damage than IHCs (Stebbins et al., 1979; Rydmarker and Nilsson, 1987; Oesterle et al., 2008). While the mechanism is not fully understood, differences in mitochondrial function and intracellular calcium homeostasis have been suggested (Sha et al., 2001; Wang et al., 2019). In contrast, our data showed that after *Pou4f3* deletion from HCs at birth, IHCs died at a faster rate than OHCs, with almost complete loss of IHCs within 1 week. A previous study showed that IHCs express a higher level of *Pou4f3* compared to their OHC counterparts (Liu et al., 2014). Therefore, the enhanced rate of IHC loss may suggest an increased dependence on *Pou4f3* to promote their survival. We unfortunately were not able to investigate the role of *Pou4f3* in IHCs at older ages due to a lack of available CreER lines.

Our data also showed that immature OHCs died at a much faster rate after *Pou4f3* deletion compared to mature OHCs. Specifically, when *Pou4f3* was deleted at P0, >50% of the OHCs were missing within 5 days. However, when the deletion was initiated at 8 weeks of age, we observed no significant OHC loss until at least 2 weeks after *Pou4f3* deletion. These findings suggest that once OHCs have matured they have a lesser dependence on *Pou4f3* for their survival or perhaps the amount of POU4F3 protein present degrades at a slower rate in adult OHCs thereby leading to a delay in the amount of time it takes for POU4F3 to become sufficiently depleted. The degradation rate of POU4F3 protein may also differ as some studies have suggested changes in HC metabolism with age (Guo et al., 2022).

Many damaging insult studies using noise, aminoglycosides and cisplatin have shown that OHC loss generally follows a basal to apical gradient with OHC loss occurring faster and/or being more pronounced in the basal turn (Leake et al., 1997; Hertzano et al., 2004, 2007; Taylor et al., 2012; Kurabi et al., 2017). Consistent with these previous findings, *Pou4f3* deletion from OHCs produced a similar base to apex gradient when the deletion occurred at juvenile or adult ages. Interestingly, there was a significant number of OHCs (20%−40%) remaining in the apex 6 weeks after *Pou4f3* deletion was induced at the juvenile or adult ages, but all HCs were gone 4 weeks after *Pou4f3* deletion was induced at birth. While we did not examine later timepoints, we suspect that these apical OHCs eventually die rather than being resistant to the effects of *Pou4f3* depletion, however, future studies will be required to confirm this. Previous studies have shown that more HCs remain in the apical turn when *Pou4f3* was mutated (using the *ddl* mutant) vs. a complete germline deletion of *Pou4f3* (Xiang et al., 2003; Pauley et al., 2008). Thus, apical HCs may be sensitive to the age and level of *Pou4f3* deletion.

The majority of SGNs (95%) are type I fibers which make synaptic connections with IHCs for sound transduction (Spoendlin, 1972; Perkins and Morest, 1975; Nienhuys and Clark, 1978). In contrast, OHCs are innervated by the type II SGN fibers (~5% of total SGNs), as well as a small percentage of type I fibers (Berglund and Ryugo, 1987; Raphael and Altschuler, 2003; Koundakjian et al., 2007; Coate et al., 2015; Elliott et al., 2021). Several studies have shown a critical period for SGN survival at neonatal ages where loss of HCs or loss of neurotrophic support caused SGN loss soon afterwards (Ernfors et al., 1995; Leake et al., 1997; Fritzsch et al., 1998; Rubel and Fritzsch, 2002; Barclay et al., 2011; Elliott et al., 2022). This period of sensitivity may occur because SGN innervation is still developing and being refined until ~ 2 weeks of age (Sobkowicz et al., 1986; Huang et al., 2012). When HCs were specifically ablated using diphtheria toxin (DT) in *Pou4f3*^*DTR*^ mice, there was significant SGN loss when DT was injected at a neonatal age (P2), but not when DT was injected at P21 (Tong et al., 2015). In our model, we observed a similar amount of SGN loss (~85%) at 4 months after *Pou4f3* deletion when both IHCs and OHCs were missing consistent with previous findings after germline *Pou4f3* deletion (Xiang et al., 2003). However, when OHCs died after *Pou4f3* was deleted at 8 weeks of age, there was a ~30%−50% loss of SGN cell bodies 4 months later. This unexpected SGN loss contrasts with the Tong et al. (2015) study. Since *Pou4f3* deletion at 8 weeks of age used *Prestin*^*CreERT*2^, IHCs were not affected and remained intact. Thus, our results suggest that *Pou4f3* may be transcribing a protein in OHCs that maintains SGN survival or inhibiting production of a protein that induces SGN cell death. In support, *Pou4f3* has been shown to positively regulate expression of the growth factors, BDNF and NT-3 (Clough et al., 2004). It is also possible that the mechanism of cell death can impact SGN survival. It is unlikely that the insertion of *loxP* sites into the *Pou4f3* locus impaired its function since our control samples were CreER-negative littermates which contained the *Pou*4*f*3^*loxP*/loxP^ allele and had similar ABR thresholds and SGN densities as wild-type mice on a similar background (Huang et al., 2013; Tong et al., 2015).

Patients with DFNA15, who have one normal copy of *Pou4f3*, are born with normal hearing, and experience progressive hearing loss with different ages of onset, most commonly occurring after adolescence (Vahava et al., 1998; Frydman et al., 2000; Weiss et al., 2003). Thus, hearing loss could be caused by insufficient levels of POU4F3 or interference of the mutant protein with the normal POU4F3, impacting protein function, stability, cellular localization, or DNA binding (Collin et al., 2008). A recent study highlighted that expression of POU4F3 encoded by missense variants was reduced compared to wild-type POU4F3 levels. Although these mutant proteins localized to nucleus, they were vulnerable to degradation. However, a frame shift variant of *Pou4f3* led to the formation of a truncated protein which primarily localized to the cytoplasm (Lee et al., 2023). In a familial case of DFNA15 where the mutation caused *Pou4f3* complete deletion, haploinsufficiency is thought to be most likely the underlying cause of hearing impairment (Freitas et al., 2014). Genetic screening for *Pou4f3* variants in a Japanese population demonstrated that individuals with mutations that produce a truncated POU4F3 showed earlier onset and slower progression of hearing loss than patients carrying different non-truncating mutations in *Pou4f3* (Kitano et al., 2017). However other studies have shown that the age of onset varied within family members who have the same genetic mutation in the *Pou4f3* gene (Pauw et al., 2008; Cui et al., 2020). Thus, while our work shows the importance of *Pou4f3* in regulating the survival and maintenance of mouse cochlear HCs at postnatal and adult ages, there is still much to learn in understanding the mechanism of DFNA15-induced hearing loss.

## Data availability statement

The original contributions presented in the study are included in the article/Supplementary material, further inquiries can be directed to the corresponding authors.

## Ethics statement

All animal work was conducted according to Institutional Animal Care and Use Committee approved protocol at Southern Illinois University School of Medicine. The study was conducted in accordance with the local legislation and institutional requirements.

## Author contributions

JS: Writing – original draft, Writing – review & editing, Formal analysis, Investigation, Methodology, Project administration, Data curation, Visualization. MR: Formal analysis, Writing – review & editing, Data curation, Methodology. BW: Conceptualization, Formal analysis, Funding acquisition, Writing – review & editing. BC: Conceptualization, Data curation, Formal analysis, Funding acquisition, Resources, Supervision, Writing – original draft, Writing – review & editing.
