# Supplementary material for: The transcription factor Pou4f3 is essential for the survival of postnatal and adult mouse cochlear hair cells and normal hearing
Source: Front Cell Neurosci. 2024 Mar 19;18:1369282. doi: 10.3389/fncel.2024.1369282 (PMC10985149; doi:10.3389/fncel.2024.1369282)
Supplement: Supplementary file 1 [file Data_Sheet_1.docx]

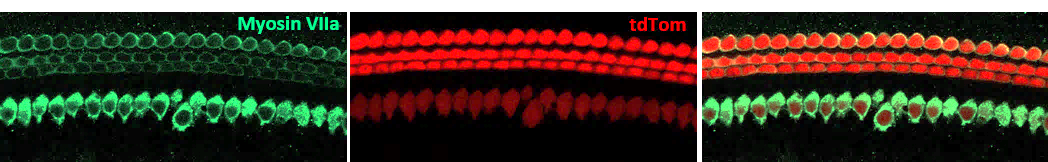


***Atoh1-*Cre*ER^TM^***

**5 days post-Tam**

**A’**

**A’’**

**A**

**B**


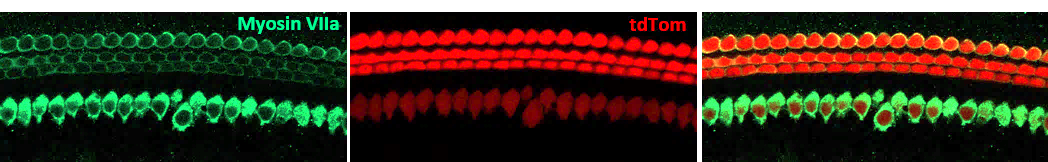


**A’**

**A’’**

**Supplemental figure 1. *Atoh1-*Cre*ER^TM^* targets the majority of OHCs and IHCs after tamoxifen induction at P0-P1.** (A-A”) Representative confocal images of cochlea from *Atoh1-CreER^TM^*:*Rosa26^tdTomato/+^* mice injected with tamoxifen at P0 and P1 and analyzed at P5. IHCs and OHCs are labeled with myosin VIIa (green) and tdTomato (red) identifies cells with CreER activity. (B) Quantification of tdTomato-positive OHC or IHCs as a percentage of total cells in the same region. Comparison across cochlear turns was done using a two-way ANOVA followed by Tukey’s post-hoc test which showed no main effects and no significant differences. Data are presented as mean ± SEM. N=4. Scale bar= 20 µm.

**B**

**A**

**1wk post-Tam**


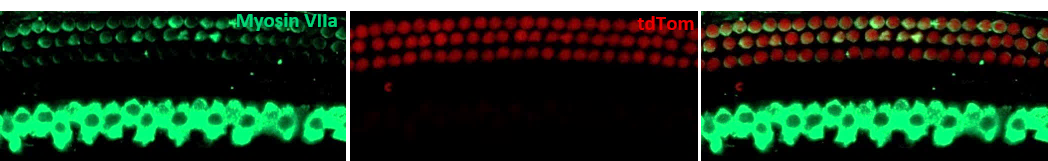


**A**

**A’**


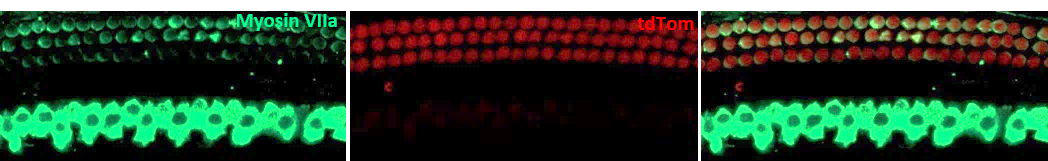


**A’’**

**A**

***Prestin^CreERT2^***

**Supplemental figure 2. *Prestin^CreERT2^* targets the majority of OHCs after tamoxifen induction at 2 weeks of age or later.** (A-A”) Representative confocal images of cochlea from *Prestin^CreERT2^*: *Rosa26^tdTomato/+^* mice injected with tamoxifen at 4 weeks (wks) of age and analyzed 1 wk later. OHCs are labeled with myosin VIIa (green) and tdTomato (red) identifies cells with CreER activity. (B) Quantification of tdTomato-positive OHCs after tamoxifen induction at P12 and P13, 4 wks or 8 wks of age as a percentage of total OHCs in the same region. There was a significant main effect of cochlear turn (F(2,18) = 4.851, p=0.0206). Asterisks indicate significant difference between cochlear turn at the respective tamoxifen induction timepoint based on a Tukey’s post-hoc test. Data are presented as mean ± SEM. N=3. *p<0.05 Scale bar= 20 µm.

There was a significant main effect of time (F(4, 10) = 29.01, p<0.0001); cochlear turn (F(2,20) = 13.88 , p=0.0002); and an interaction between time and cochlear turn (F(8, 20) = 3.68, p=0.0085). Differences from control within each cochlear turn are indicated by the asterisks based on a Tukey’s-corrected post-hoc test.

**Supplemental Table 1. Primer sequences used for real-time qPCR.**

| **Gene name** | **Orientation** | **Primer Sequence** | **Reference** |
| --- | --- | --- | --- |
| ***Bcl2*** | **F** | **GGA CTT GAA GTGCCA TTG GT** | **Viant et al 2017 J Exp Med** |
|  | **R** | **CGG TAG CGA CGA GAGAAG TC** |  |
| ***Bcl6*** | **F** | **CCT GCA ACT GGA AGA AGT ATA AG** | **Duy et al 2010, J Exp Med** |
|  | **R** | **AGT ATG GAG GCA CAT CTC TGT AT** |  |
| ***Bcl-XL*** | **F** | **AGGATACAGCTGGAGTCAG** | **Valks et al, 2003 J. Biol Chem** |
|  | **R** | **TCTCCTTGTCTACGCTTTCC** |  |
| ***Gfi1*** | **F** | **AGGAACGCAGCTTTGACTGT** | **Ichiyama et al 2009, International Immunology** |
|  | **R** | **GATGAGCTTTGCACACTGGA** |  |
| ***Lhx3*** | **F** | **TGCTGCTATTTCCGACAGGGC** | **Tian et al, 2008 Dev Dyn** |
|  | **R** | **GAACCAACAGGTAGCCAAGATCC** |  |
| ***p53*** | **F** | **AGAGACCGCCGTACAGAAGA** | **Mazoochi et al 2009 Mol Hum Rep** |
|  | **R** | **GCATGGGCATCCTTTAACTC** |  |
| ***Stat3*** | **F** | **GTGGAGAACCTCCAGGACGA** | **Xu et al 2018, Infection and Drug Resistance** |
|  | **R** | **GCCAGCTCACTCACAATGCT** |  |
| ***Rpl19*** | **F** | **GGTCTGGTTGGATCCCAATG** | **Korrapati, 2013 PLOS One** |
|  | **R** | **CCCGGGAATGGACAGTCA** |  |
| ***Bax*** | **F** | **GCG TGG TTG CCC TCT TCT ACT TTG** | **Medearis et al 2011, Invest Ophthalmol Vis Sci** |
|  | **R** | **AGT CCA GTG TCC AGC CCA TGA TG** |  |
| ***Bak1*** | **F** | **AAG GTG GGC TGC GAT GAG TCC** | **Medearis et al 2011, Invest Ophthalmol Vis Sci** |
|  | **R** | **GGG TCT CCT GTT CCT GCT GGT G** |  |
| ***Bad*** | **F** | **GGA AGA CGC TAG TGC TAC AG** | **Wildhaber et al 2002, Pediatr Surg Intl** |
|  | **R** | **GAG CCT CCT TTG CCC AAG TT** |  |

**Supplemental Table 2**

Supplemental Table 2A:

| **Cochlear turn** | **% OHC loss (control)** | **% OHC loss (*Atoh1-Pou4f3*cKO)** | | | |
| --- | --- | --- | --- | --- | --- |
|  |  | **5 days**  **post-Tam** | **1 wk**  **post-Tam** | **2 wks**  **post-Tam** | **4 wks**  **post-Tam** |
| **Apex** | **-0.0 ± 2.4** | **64.8 ± 4.2** | **80.2 ± 0.9** | **78.9 ± 5.7** | **95.2 ± 1.0^###^** |
| **Middle** | **2.8 ± 1.5** | **55.9 ± 1.2** | **36.8 ± 1.6***** | **91.8 ± 3.3^###, $$$$^** | **96.7 ± 2.1^####, $$$$^** |
| **Base** | **0.0 ± 2.7** | **58.4 ± 15.9** | **51.7 ± 2.2***** | **97.4 ± 2.2^*,####, $$$$^** | **97.5 ± 1.3 ^####, $$$$^** |

Supplemental Table 2B:

| **Cochlear turn** | **% IHC loss (control)** | **% IHC loss (*Atoh1-Pou4f3*cKO)** | | | |
| --- | --- | --- | --- | --- | --- |
|  |  | **5 days**  **post-Tam** | **1 wk**  **post-Tam** | **2 wks**  **post-Tam** | **4 wks**  **post-Tam** |
| **Apex** | **0.1 ± 3.3** | **44.7 ± 16.4** | **98.2 ± 1.8^####^** | **98.1 ± 1.9^####^** | **99.1 ± 0.9^####^** |
| **Middle** | **0.0 ± 0.0** | **33.6 ± 17.4** | **96.6 ± 2.3^####^** | **97.4 ± 1.5^####^** | **99.1 ± 0.9^####^** |
| **Base** | **0.0 ± 3.5** | **53.4 ± 10.5^˄^** | **93.9 ± 3.5^##^** | **98.1 ± 1.0^##^** | **90.4 ± 4.8^##^** |

**Supplemental Table 2. Statistical comparisons of HC loss across cochlear turns after *Pou4f3* deletion at neonatal age.** Data from Figure 1F (percentage of OHC loss) and 1G (percentage of IHC loss) are presented as mean ± SEM. Comparison of OHC and IHC loss across time post-tamoxifen was done using a Tukey’s post-hoc test. #represents statistical significance from the 5-days post-tamoxifen timepoint within the same cochlear turn. $represents statistical significance from the 1 week (wk) post-tamoxifen timepoint within the same cochlear turn. A second Tukey’s post-hoc test was used to compare OHC or IHC loss across cochlear turns within the same genotype and same analysis timepoint. *represents statistical significance from the apical turn within the same analysis timepoint. A single symbol represents p<0.05, two symbols represents p<0.01, three symbols represents p<0.001, and four symbols represent p<0.0001.

**Supplemental Table 3**

| **Cochlear turn** | **% OHC loss (control)** | **% OHC loss (*Prestin-Pou4f3*cKO)** | | | |
| --- | --- | --- | --- | --- | --- |
|  |  | **1 wk**  **post-Tam** | **2 wks**  **post-Tam** | **4 wks**  **post-Tam** | **6 wks**  **post-Tam** |
| **Apex** | **0.0 ± 4.8** | **24.2 ± 10.1** | **26.5± 5.1** | **41.2 ± 10.6** | **61.5 ± 5.3^##^** |
| **Middle** | **0.0 ± 1.1** | **47.6 ± 16.8** | **69.3 ± 4.5 ^***^** | **76.1 ± 2.9^**^** | **97.5 ± 1.7^**, ####, $^** |
| **Base** | **0.0 ± 11.5** | **84.9 ± 2.9^****, ˄˄^** | **85.0 ± 3.9^****^** | **90.6 ± 7.0^***^** | **97.2 ± 1.5^**^** |

**Supplemental Table 3. Statistical comparisons of OHC loss across cochlear turns after *Pou4f3* deletion at hearing onset.** Data from Figure 2F are presented as mean ± SEM. Comparison of OHC loss across time post-tamoxifen was done using a Tukey’s post-hoc test. #represents statistical significance from the 1 week (wk) post-tamoxifen timepoint within the same cochlear turn. $represents statistical significance from the 2 wks post-tamoxifen timepoint within the same cochlear turn. A second Tukey’s post-hoc test was used to compare OHC loss across cochlear turns within the same genotype and same analysis timepoint. *represents statistical significance from the apical turn within the same analysis timepoint. ^˄^represents statistical significance from the middle turn within the same analysis timepoint. A single symbol represents p<0.05, two symbols represents p<0.01, three symbols represents p<0.001, and four symbols represent p<0.0001.

**Supplemental Table 4**

| **Cochlear turn** | **OHC loss (control)** | **% OHC loss (*Prestin-Pou4f3*cKO)** | | | |
| --- | --- | --- | --- | --- | --- |
|  |  | **1 wk**  **post-Tam** | **2 wks**  **post-Tam** | **4 wks**  **post-Tam** | **6 wks**  **post-Tam** |
| **Apex** | **0.0 ± 5.0** | **10.2 ± 4.1** | **15.1 ± 3.4** | **55.2 ± 13.4^##, $^** | **82.1 ± 5.4^####, $$$^** |
| **Middle** | **0.0 ± 1.7** | **30.3 ± 12.9** | **55.2 ± 13.4^**^** | **85.8 ± 7.7 ^*, ###^** | **90.4 ± 9.6 ^####^** |
| **Base** | **0.0 ± 9.8** | **85.6 ± 2.9^****, ˄˄˄^** | **85.8 ± 7.7^****, ˄^** | **86.3 ± 4.1^*^** | **88.3 ± 10.5** |

**Supplemental Table 4. Statistical comparisons of OHC loss across cochlear turns after *Pou4f3* deletion at 4 weeks (wks) of age.** Data from Figure 3F are presented as mean ± SEM. Comparison of OHC loss across time post-tamoxifen was done using a Tukey’s post-hoc test. # represents statistical significance from 1 wk post-tamoxifen timepoint within the same cochlear turn. $represents statistical significance from the 2 wks post-tamoxifen timepoint within the same cochlear turn. A second Tukey’s post-hoc test was used to compare OHC loss across cochlear turns within the same genotype and same analysis timepoint. *represents statistical significance from the apical turn within same analysis timepoint. ^˄^represents statistical significance from the middle turn within same analysis timepoint. A single symbol represents p<0.05, two symbols represents p<0.01, three symbols represents p<0.001, and four symbols represent p<0.0001.

**Supplemental Table 5**

| **Cochlear turn** | **OHC loss (control)** | **% OHC loss (*Prestin-Pou4f3*cKO)** | | | |
| --- | --- | --- | --- | --- | --- |
|  |  | **1 wk**  **post-Tam** | **2 wks**  **post-Tam** | **4 wks**  **post-Tam** | **6 wks**  **post-Tam** |
| **Apex** | **-0.0 ± 2.8** | **3.2 ± 2.2** | **27.5 ± 5.6** | **58.1 ± 15.8 ^##^** | **72.8 ± 10.1 ^####^** |
| **Middle** | **0.0 ± 6.4** | **4.4 ± 6.7** | **31.0 ± 6.8** | **81.0 ± 17.4 ^###^** | **94.7 ± 1.0 ^###, $^** |
| **Base** | **0.0 ± 15.7** | **9.6 ± 13.4** | **84.5 ± 9.8^****, ˄˄˄^** | **98.4 ± 1.1^**^** | **92.3 ± 5.7** |

**Supplemental Table 5. Statistical comparisons of OHC loss across cochlear turns after *Pou4f3* deletion from OHCs at 8 weeks age.** Data from Figure 4F are presented as mean ± SEM. Comparison of OHC loss across time post-tamoxifen was done using a Tukey’s post-hoc test. #represents statistical significance from the 1 week (wk) post-tamoxifen timepoint within the same cochlear turn. $represents statistical significance from the 2 wk post-tamoxifen timepoint within the same cochlear turn. A second Tukey’s post-hoc test was used to compare OHC loss across cochlear turns within the same genotype and same analysis timepoint. *represents statistical significance from the apical turn within same analysis timepoint. ^˄^represents statistical significance from the middle turn within same analysis timepoint. A single symbol represents p<0.05, two symbols represents p<0.01, three symbols represents p<0.001, and four symbols represent p<0.0001.
